# Immunogenic Effect of Hyperthermia on Enhancing Radiotherapeutic Efficacy

**DOI:** 10.3390/ijms19092795

**Published:** 2018-09-17

**Authors:** Sungmin Lee, Beomseok Son, Gaeul Park, Hyunwoo Kim, Hyunkoo Kang, Jaewan Jeon, HyeSook Youn, BuHyun Youn

**Affiliations:** 1 Department of Integrated Biological Science, Pusan National University, Busan 46241, Korea; smlee1048@gmail.com (S.L.); beomseok15@gmail.com (B.S.); gaeulpark91@gmail.com (G.P.); harlemkim@gmail.com (H.K.); kanghk94@gmail.com (H.K.); jjwej1066@gmail.com (J.J.); 2 Department of Integrative Bioscience and Biotechnology, Sejong University, Seoul 05006, Korea; 3 Department of Biological Sciences, Pusan National University, Busan 46241, Korea

**Keywords:** hyperthermia, immunotherapy, radiotherapy, combination therapy

## Abstract

Hyperthermia is a cancer treatment where tumor tissue is heated to around 40 °C. Hyperthermia shows both cancer cell cytotoxicity and immune response stimulation via immune cell activation. Immunogenic responses encompass the innate and adaptive immune systems, involving the activation of macrophages, natural killer cells, dendritic cells, and T cells. Moreover, hyperthermia is commonly used in combination with different treatment modalities, such as radiotherapy and chemotherapy, for better clinical outcomes. In this review, we will focus on hyperthermia-induced immunogenic effects and molecular events to improve radiotherapy efficacy. The beneficial potential of integrating radiotherapy with hyperthermia is also discussed.

## 1. Current State of Radiotherapy and Immunotherapy

Radiotherapy is a widely used, well-established anti-tumor treatment that has shown significant clinical outcomes. Utilizing ionizing radiation, radiotherapy generates excessive oxidative stress and induces DNA damage, such as single or double DNA strand breaks, and tumor cell deaths [1]. As radiation can penetrate the body, and can be accurately limited to the depth of interest, radiotherapy is a non-invasive and spatially specific strategy compared to other anti-tumor therapies [2]. However, some tumor cells can be radioresistant, showing resistance to radiation-induced oxidative stress and DNA damage-induced cell death through various intracellular pathways [3,4]. Although increased radiation dose is more likely to induce tumor cell deaths, an excessively high radiation dose can induce damage in adjacent normal tissue and related side effects. For this reason, several radiosensitization strategies have been developed for better therapeutic efficacy. Mostly, combinations of radiosensitizing chemotherapy and radiotherapy are used, providing better post-therapy outcomes [5]. In addition, immunotherapy, which elevates systemic immunity against tumor cells, has shown radiosensitizing effects and better therapeutic outcomes [6]. However, radioresistance has not been fully overcome, and current studies focus on novel strategies for enhancing therapeutic efficacy.

Immunotherapy enhances the immune cells’ ability to recognize and target tumor cells, leading to their elimination. The advantages of immunotherapy include high anti-tumor specificity and minimal side effects by utilizing the patient’s own immune system [7]. Current immunotherapies focus on suppressing tumor cell evasion using antibodies that inhibit immune checkpoint molecules, including cytotoxic T-lymphocyte-associated antigen 4 (CTLA-4), programmed death 1 (PD-1), and programmed death-ligand 1 (PD-L1) [8]. Antibodies specific for these molecules are called immune checkpoint blockades, and include anti-CTLA-4 (ipilimumab) and anti-PD-1 (nivolumab and pembrolizumab) antibodies, which are United States Food and Drug Administration (FDA) approved for clinical treatment with significant therapeutic outcomes [8]. Furthermore, current studies covering the radiosensitization effect of immunotherapy suggest the potential of combining immunotherapy and radiotherapy [9]. However, the biggest obstacle in immunotherapy application is the relatively low efficacy, and difficulty in achieving tumor cell-specific immunogenicity, compared to other anti-tumor therapies [10]. Although immune checkpoint blockade antibodies should directly bind to the tumor cell surface for its effect, recent studies suggested that the limitation of immunotherapy is the inefficient delivery to tumor sites [11]. This was also supported by other studies that suggested a novel immune checkpointblockade delivery system, through conjugation with nanoparticles and homing molecules, for efficient delivery. However, the therapeutic significance was still low, and current immunotherapies need further modification for clinically meaningful immunogenic effects.

To better induce immunity against tumor cells, immunogenic therapeutic adjuvants were suggested, some of which showed significantly increased therapeutic efficacy with low normal tissue damage [12]. Recent studies also applied these immunogenic adjuvants to tumor cell sensitization against therapies. For example, platinum-based chemotherapies including cisplatin, carboplatin, and oxaliplatin are widely used as anti-tumor treatments, and show immunogenic effects through cell death induction and the release of death-associated molecular patterns, which activate pro-inflammatory signaling pathways [13]. Similarly, the immunogenic effects of other cytotoxic chemicals also supported this phenomenon [14,15]. However, these chemicals also showed cytotoxicity to normal tissues and induced severe side effects, which slows their clinical application [16,17]. Recently, immunogenic biological derivatives were suggested as an immunogenic approach with fewer side effects. Biological derivatives, such as peptides, glycosides, and natural products, have shown significant immunogenicity through immune cell activation and tumor cell deaths [18,19,20]. Although these studies supported the crucial role and promising potential of immunogenic anti-tumor therapies, neither these adjuvants nor immunotherapy could fulfill the demanding therapeutic efficacy and tumor cell-specific delivery, leaving them as major obstacles to overcome.

Alternatively, hyperthermia was recently suggested as an immunogenic treatment with spatial specificity and high efficacy [21]. And the modification of temperature and treatment duration can control the biological effects of hyperthermia treatment [22]. Furthermore, increased immunity by hyperthermia resulted in tumor cell sensitization against radiotherapy [23]. Although the significant anti-tumor effects of hyperthermia combined with radiotherapy were recently reported, a clinical protocol for the best outcome has not been devised yet. This suggested the need for a deeper understanding of the immunogenic effects of hyperthermia as an anti-tumor adjuvant for clinical applications with better outcomes. In this review, we summarize the current studies on the hyperthermia-induced immunogenicity, and the underlying molecular mechanisms for combining hyperthermia and radiotherapy. In addition, we also provide promising clinical significance for applying hyperthermia as an adjuvant to overcome radioresistance and enhance therapeutic efficacy.

## 2. The Immunogenic Effect of Hyperthermia

Hyperthermia treatment involves increasing the target site temperature to induce thermic stress, with an average temperature around 40 °C (39–43 °C, dependent on therapeutic strategies). In previous studies, hyperthermia was typically reported to modulate local pro-inflammatory responses, immune activation, cell death, and microenvironmental changes in the target sites, which were mostly mediated by specific chaperone proteins called heat shock proteins (Hsps) [24]. Highamounts of Hsps are expressed upon environmental stress, including heat shock, oxidative stress, and chemical stress [25]. Hsps prevent and reverse heat-induced protein misfolding, and recover heat-induced cellular damages through chaperone activity. In addition, some Hsps are extracellularly exposed and function as signaling molecules to induce a heat shock response in adjacent cells. Transcriptional heat shock response is mediated by the heat shock transcription factor (HSF) via the “chaperone titration model” [26]. In this model, HSF is bound to Hsp70 and Hsp90 in the cytosol in non-stressed circumstances. Upon heat shock, Hsp70 and Hsp90 dissociate from HSF to act as chaperones. The free HSFs form trimer and move to the nucleus where they have transcription factor activity. As responses after hyperthermia treatment are also mediated by Hsps and HSF, understanding of Hsp and HSF molecular mechanisms can explain the biological role of hyperthermia.

**Figure 1:**
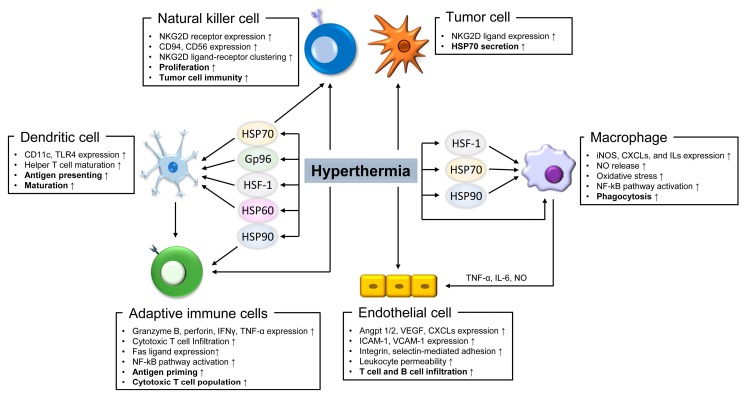
Hyperthermia-induced immunogenicity in immune cells. Diagram summarizes the effects of hyperthermia and heat shock proteins (Hsps) in immune response enhancement in macrophage, dendritic cell, natural killer cell, endothelial cell, adaptive immune cells, and tumor cell. VEGF: vascular endothelial growth factor; iNOS: inducible nitric oxide (NO) synthase; CXCLs: chemokine (C-C motif) ligands; ICAM-1: intracellular adhesion molecule 1; VCAM-1: vascular cell adhesion molecule 1; TNF-α: tumor necrosis factor α; NKG2D: natural killer lectin-like receptor gene 2D. HSP: heat shock proteins; TLR: toll-like receptor; ILs: interleukins. The arrows indicate the upregulation effect by hyperthermia.

Among the various biological effects of hyperthermia, the immunogenic regulation of cancer progression has been widely studied, and showed promising outcomes through increasing therapeutic efficacy [27,28]. The immunogenicity of hyperthermia has been classically known. Upon infection, adjacent innate immune cells sense pathogen-associated molecular patterns such as lipopolysaccharide and exogenous nucleotides, and secrete cytokines, including interleukin (IL) -1, IL-6, and tumor necrosis factor α (TNF-α). These cytokines circulate in the blood, stimulate the hypothalamus, and activate the COX-2- or RANKL-PGE_2_ signaling pathways to produce a fever [29,30]. Accordingly, a fever increases systemic temperature and induces pro-inflammatory signaling in the immune cells. Herein, we have summarized the effects of hyperthermia and heat shock responsive factors on enhancing immune cell immunogenicity, which supports the involvement of hyperthermia in increasing the efficacy of anti-tumor therapy (Figure 1).

### 2.1. Innate Immune Cells

In an immune response, innate immune cells respond to the fever first, which increases their activity. Previous studies suggested that hyperthermia activates macrophages through Hsp-mediated mechanisms, as well as the immunogenic effects of hyperthermia. It was reported that heat shock increased the expression of inducible nitric oxide (NO) synthase (iNOS), chemokine (C-C motif) ligands (CXCLs), and IL and NO release, which activate the functions of macrophages [31,32]. In addition, heat shock enhanced the nuclear factor kappa beta (NF-κB) signaling pathway through induction of IκBα degradation [33]. These effects were also validated by studies showing that heat shock increased macrophage phagocytic activity [34,35,36]. Upon heat stress, HSF1 induces the genes involved in macrophage activation, as well as Hsp expression [37,38]. Various Hsps are involved in the regulation of macrophage activation, which also supports the role of hyperthermia. Specifically, Hsp70 was reportedly related to heat shock-induced macrophage activation, iNOS expression, NF-κB signaling activation, and enhanced phagocytosis [36,39,40,41,42]. Studies on Hsp90 inhibition suggested that Hsp90 controls macrophage activation, NADPH oxidase (NOX) expression, oxidative stress, NF-κB signaling activation, and phagocytosis [36,43,44,45]. Although some studies reported the macrophage inactivation effect of Hsp90 inhibition, the Hsp90 inhibitors used in these studies also reportedly increased Hsp70 expression, causing complex molecular events to occur [43,44,46]. Moreover, Hsp23, Hsp27, and Hsp40, were also involved in macrophage activation, which supports the important role of hyperthermia [36,47,48,49]. Conversely, some studies reported the heat shock-induced inactivation and decreased survival of macrophages [50,51,52]. These studies showed that prolonged and strong heat shock could result in immune response defects and tissue damage, suggesting the importance of hyperthermia intensity and duration.

Dendritic cells (DCs) are a type of innate immune cell which presents antigens to adaptive immune cells to create immunity. The antigen presentation by DCs is significant in generation of immunity against tumor cells. In previous studies, both intense and mild heat shock induced DC maturation and inflammatory signaling activation [53,54]. Other studies also supported that heat shock induced mature CD11c^+^ DCs through HSF1-, Hsp70-, and toll-like receptor (TLR)-dependent manners [55,56,57,58]. Previous studies also suggested the extraordinary role of Hsp70 in DC activation. Hsp70 is secreted by adherent tumor cells upon heat shock reaction and acts as a DC antigen, which increases immunity against tumor cells through TLR4 in DCs [59,60]. This role of Hsp70 was reported in glioblastoma, hepatocarcinoma, and melanoma cells, and in some clinical studies [61,62,63,64,65]. DCs also reportedly expressed Hsp90 at high temperature, and showed accelerated maturation [66]. The activating role of hyperthermia through Hsp90 was validated in DCs via specific Hsp90 inhibition interfering with the DCs-mediated CD4^+^ T cell maturation [67,68]. Similar to Hsp70, Hsp90 was also reported to increase immunogenicity against tumor cells by forming complexes of tumor antigen peptide to donor cells [69,70,71,72]. Furthermore, studies about the involvement of Hsp60, gp96 (a member of the Hsp90 family), and Hsc73 in DC activation, supported the hyperthermia-induced immunogenicity of DCs [73,74,75,76,77,78]. These studies show that hyperthermia increases the proliferation, maturation, and antigen presentation of DCs, which may lead to adaptive immune response activation.

Natural killer (NK) cells, the most effective cells involved in tumor growth and recurrence inhibition, were reportedly activated by hyperthermia through enhanced cytotoxicity and recruitment to tumor sites in vivo [79,80,81]. Increased NK cell cytotoxicity was dependent on thermal stimulation-induced clustering of natural killer lectin-like receptor gene 2D (NKG2D) ligand-receptors on tumor and NK cells. This clustering was mediated by hyperthermia-induced Hsp70 expression, which led to NKG2D ligand expression in various types of cancer cells, including colon, lung, cervical, and skin cancer [82,83,84]. Hsp70 could also stimulate NK cell proliferation and activity through the expression of NKG2D, CD94, and CD56 in NK cell membranes [85,86,87]. These studies suggested that hyperthermia increased NK cell immunity through dual stimulation of tumor and NK cells. Hsp90 was studied as another mediator in the induction of NK cell activation using Hsp90 inhibitors [88,89]. Moreover, Hsp72, gp96, and HSF-1 were also reportedly involved in the enhancement of NK cell activity [90,91,92]. In summary, hyperthermia can increase NK cell immunity against tumor cells by activating NK cells, and increasing tumor cell recognition by NK cells.

### 2.2. Immune Cell Infiltration.

After activation of innate immune cells, naïve leukocytes and lymphocytes are attracted, and infiltrate into the tissue from blood or lymph to interact with antigen-presenting cells. Previous studies have suggested that hyperthermia may help immune cell infiltration through increased endothelial cell activation and permeability. In mild hyperthermia treatment, endothelial cells showed increased expression of angiopoietin (Angpt) 1 and 2, which stimulated vasculogenesis and immune cell infiltration [93]. Although the role of vascular endothelial growth factor (VEGF) in regulating vessel permeability is controversial, hyperthermia was reported to induce VEGF expression in endothelial cells [93,94,95]. We interpreted that hyperthermia enhanced immune cell infiltration through an overall induction of vascular network formation. The roles of Hsps in endothelial cell activation also supported the significant involvement of hyperthermia. Hsp70 expressed post-hyperthermia treatment could move to the extracellular environment [96]. Increased extracellular Hsp70 stimulated normal macrophages to secrete the cytokines TNF-α, IL-6, and NO, which enhanced endothelial wall activation and permeability [97,98,99,100]. These cytokines induced the expression of chemokines including CXCL1, CXCL2, and CXCL10, and adhesion molecules including intracellular adhesion molecule 1 (ICAM-1) and vascular cell adhesion molecule 1 (VCAM-1) in endothelial cells, which modulate the interaction with infiltrating immune cells [101,102,103]. A study examining the role of hyperthermia in increasing endothelial cell adhesion to immune cells directly supported these events [104]. Endothelial cell activation in the central nerve system played a major role in fever generation during pro-inflammatory situations, and hyperthermic immunogenicity could be maintained through endothelial cell activation [105,106]. Hyperthermia also increased the integrin- and selectin-mediated adhesion of lymphocytes to the endothelium [107,108]. Endothelial cell activation and increased immune cell recruitment result in the formation of anti-tumoral microenvironments. This was observed in a previous study where hyperthermia increased the number of infiltrating T and B cells in in vitro and tumor xenograft models [109].

### 2.3. Adaptive Immune Cells

Cytotoxic T cell activation upon hyperthermia has been supported by previous studies, which provided evidence for the hyperthermia-induced immunogenicity of adaptive immunity. One study reported that hyperthermia treatment induced the expression of granzyme B, perforin, and interferon γ (IFNγ), and increased the cytotoxic activity of cytotoxic T cells [110]. Furthermore, the study suggested that mild hyperthermia (39 °C) showed the best immunogenic effects compared with hyperthermia at other temperatures (~41 °C). This supported the importance of modifying hyperthermia treatment strategies for the best effects. Previous studies reported that hyperthermia induced lymphocyte migration from circulation to the peripheral lymph nodes, local tissue, and even tumor sites in in vivo mouse models [111,112]. Heat shock responsive proteins were also involved in regulating T cell immunity upon hyperthermia. Hyperthermia-induced HSF directly promoted Fas ligand expression and immunity against Fas-bearing target cells both in vitro and in vivo [113,114]. In the context of Hsp, Hsp70 acted as self-epitopes and activated T cells through the induction of CD69 and cytokines, including IFNγ and TNF-α [115]. Hsp90 and Hsp27 also reportedly acted as antigens to induce immunity against myeloma cells, and increased active antigen-specific cytotoxic T cells [116]. Enhancement of hyperthermia-induced Hsp-specific immunity is crucial in tumor progression regulation because tumor cells naturally showed high Hsp expression. The role of Hsps in regulation of signaling pathways was also reported. Hsp90 was involved in TNF-α expression, NF-κB signaling pathway activation, and the pro-inflammatory signaling pathway in cytotoxic T cells [117]. Gp96 showed cross-protective roles in cytotoxic T cells, and had increased activity against tumor cells [118,119]. Gp96 also induced antigen-specific T cell activation through changing the regulatory T cell/cytotoxic T cell ratio by reducing the regulatory T cell population [120]. Gp96 and Hsp80 bound to CD91 on antigen presenting cells, and mediated the priming of antigen presenting cells to T helper cells [121]. Similarly, Hsp110 and gp170 formed complexes with antigens and enhanced antigen presentation to T cells [122]. These studies showed that hyperthermia treatment increased immunogenicity through regulation of cytotoxic T cell population and activity.

Although a few studies suggested the effects of hyperthermia on B cell activation, hyperthermia was also involved in B cell activation. Hyperthermia treatment induced B cells to express TLR9 through activation of the extracellular signal-regulated kinase (ERK) and NF-κB signaling pathways, which activated B cell-mediated immune responses [123]. Hyperthermia-induced HSF1 promoted B cell proliferation and activation [124,125]. Hsp90 increased total B cell proliferation, and activated their antigen presentation via B cell-antigen complex formation [126,127]. Hsp60 protected B cells from apoptosis and increased their survival through the TLR4 signaling pathway [128]. Apg-2, a member of the Hsp110 family, induced the proliferation of, and reduced the oxidative stress in B cells [129]. Taken together, these studies supported that the hyperthermia-induced immunogenic effect could be mediated by adaptive immune cell activation.

## 3. Molecular Mechanisms of Immunogenicity by Hyperthermia

Attempts to combine radiotherapy and hyperthermia for tumor cell radiosensitization were reported in the 1980s and 1990s [130,131]. However, utilizing hyperthermia for radiosensitization was not widely used due to the difficulty of achieving delicate target site temperature regulation with past hyperthermia technologies, and ignorance of the adequate temperature for hyperthermia treatment [132]. Before the 2000s, hyperthermia treatment was delivered through invasive intraluminal- and interstitial-hyperthermia [133,134]. Invasive hyperthermia treatments were less safe because of the need to penetrate a heating node into the target site. As the hyperthermia technology and equipment developed, various types of non-invasive hyperthermia treatment methods and according outcomes were suggested in recent studies [135]. In recent decades, hyperthermia was delivered through radiofrequency-, microwave-, and electromagnetic-capacitive hyperthermia, with highly sensitive temperature control capacity [136,137,138]. The safety of non-invasive hyperthermia treatment was proven in recent studies [139,140]. Moreover, the immunogenic treatment, which increased the radiation therapy efficacy, suggests the possible application of hyperthermia for radiosensitization, and this hyperthermia-induced radiosensitization was validated by many studies [141,142,143,144]. Focusing on the immunogenicity of hyperthermia, we summarized the current studies examining the molecular events that occur during the combination of hyperthermia treatment and radiotherapy, to understand the radiosensitization effects of hyperthermia (Table 1).

### 3.1. DNA Repair

Radiation generates DNA damage through strong oxidative stress, which induces single or double strand DNA breaks. DNA damage is sensed by DNA damage response proteins, including DNA-dependent protein kinase (DNA-PK), γH2AX, MRE11, and ataxia telangiectasia mutated (ATM), which cause either DNA repair or apoptosis depending on the damage severity. Increased radiation-induced tumor cell apoptosis could activate adjacent immune cells by providing an immunogenic microenvironment [145]. A previous study suggested that DNA-PK activity was inhibited by hyperthermia, and maintained pro-apoptotic signaling by combining hyperthermia and radiation therapies in a hepatoma cell line [142]. This effect of hyperthermia was confirmed by studies that showed hyperthermia-induced γH2AX foci increase, and steady DNA damage responses were mediated by DNA-PK [146]. Hyperthermia-induced inhibition of MRE11, another DNA damage response protein, was also reported to induce radiosensitization, which was correlated with MRE11 expression [147,148]. Hyperthermia also induced MRE11 translocation toward the cytoplasm, and inhibited complex formation with Rad50 and NBS1, which increased the number of unrepaired DNA breaks and caused pro-apoptotic signaling pathway activation [149,150]. Additionally, other DNA repair proteins, including H2AX, Rad51, breast cancer gene 2 (BRCA2), and Ku70, were inhibited by hyperthermia and shown to have roles in hyperthermia-induced radiosensitization [151,152,153,154]. Furthermore, one study even suggested the possible application of hyperthermia for sensitizing tumor cells against genotoxic chemotherapy [155]. From these studies, we can conclude that combining hyperthermia and radiotherapy enhances the genomic instability of tumor cells.

### 3.2. Cell Cycle Arrest and Apoptosis

Sustained hyperthermia-induced DNA damage, due to inhibited DNA repair, induced the pro-apoptotic signaling pathway [156]. P53 was crucially involved in mediating the signaling to late DNA damage responses, even when combining hyperthermia and radiotherapy [157]. One of the late DNA damage responses, cell cycle progression, was inhibited. Hyperthermia enhanced radiation-induced cell cycle arrest in the G_2_ and M phases through intracellular accumulation of cyclin B1, which caused tumor cell apoptosis [158,159]. Other studies also suggested that combining hyperthermia and radiotherapy resulted in increased radiosensitive G_2_ phase cells and high motility group box protein B1 (HMGB1) release, acting as a danger signal, which further stimulated immune responses [160,161]. Studies examining the roles of Hsp70 and Hsp90 expression in cell cycle arrest during combination therapy, suggested the direct involvement of hyperthermia [162,163]. As another late DNA damage response, hyperthermia-induced p53 activated pro-apoptotic signaling proteins and caspases, which sensitized tumor cells to radiotherapy in in vitro and in vivo models [164,165]. Pro-apoptotic signaling activation also occurred by Bax/Bcl-2 ratio regulation via transcriptional regulation [166]. Moreover, hyperthermia could reduce the radiation-induced activation of survival pathways, including the NF-κB signaling pathway through IκB kinase inhibition, and the AKT-S6K pathway [167,168]. As the targeting of the NF-κB and PI3K-AKT-mTOR signaling pathways has been crucial to overcoming tumor cell radioresistance, hyperthermia also may sensitize tumor cells by mediating survival signaling inhibition [169,170].

### 3.3. Heat Shock Response

As the general regulator of hyperthermic stimulation, Hsps were upregulated and acted as signaling molecules and transcription factors upon heat shock. We found that some studies describing the role of Hsps in hyperthermia-induced radiosensitization suggested Hsps as the mediator of molecular events. Hyperthermia-induced Hsp72 expression increased the radio- and chemo-sensitivity of prostate cancer cells through the suppression of survival, MAPK, and NK-κB pathways [171]. This effect of combination therapy was also validated in an in vivo mouse prostate cancer model [172]. Hyperthermia also increased extracellular Hsps during tumor necrosis, which induced immunity against tumor cells [173]. However, some studies reported the roles of Hsp70 in radioresistance and Hsp72 in etoposide-induced apoptosis resistance [174,175]. This discrepancy suggested that hyperthermia-induced radiosensitization was partially dependent on Hsp expression. Taken together, these studies insisted that the underlying molecular mechanisms that mediate hyperthermia-induced radiosensitization are still elusive. Further molecular studies will be a key to the therapeutic optimization of hyperthermia as a radiosensitizing strategy.

### 3.4. Abscopal Effect

Radiation-induced immunogenicity displays the abscopal effect, a phenomenon in which local radiotherapy at a distance from a tumor site suppressed tumor growth and progression. Although the precise mechanism of the abscopal effect is not fully understood, it may be mediated by immune system activation [176,177]. Current studies support that hyperthermia-induced immunogenicity could enhance the therapeutic efficacy of radiotherapy through the abscopal effect. It was reported that magnet-mediated hyperthermia could improve abscopal antitumor effects, and stimulate significant endogenous immune responses in sarcoma-bearing rats, by improving the CD4^+^/CD8^+^ T cell ratio [178]. And it was also reported that combining intratumoral DC injection and modulated electro-hyperthermia, exerted a strong abscopal effect against distant non-treated tumors [179]. These reports showed that hyperthermia can exaggerate the radiation-induced abscopal cytotoxicity of tumor cells and enhance radiotherapy efficacy through immunogenic effects, which could be applied to combination therapy in the future.

## 4. Clinical Significance of the Immunogenic Effect of Hyperthermia

Based on the high potential shown when combining immunogenic hyperthermia and radiotherapy as an anti-tumor treatment, this combination therapy has been used in clinical research to enhance therapeutic efficacy. Current research reports that hyperthermia sensitized various tumor cells, which gave better outcomes in cancer patients. In this review, we summarized current studies on therapeutic strategies used to gain a deeper understanding of the clinical application of hyperthermia and radiation combination therapy. We noticed that the differing treatment schedules between studies determined the clinical radiosensitization effect of hyperthermia. In cervical cancer patients, only concurrent delivery of hyperthermia and radiotherapy showed significantly improved outcomes with negligible adjacent normal tissue damage [180]. In the same study, the radiosensitization effect diminished over time post-treatment, with the effect being significantly reduced 30 min after hyperthermia treatment. The effects of simultaneous hyperthermia treatment (a less than 30-min interval) were reported in superficial, breast, and cervical cancers [181,182,183,184]. Moreover, one study also suggested the suppression of lung cancer metastasis by concurrently combining hyperthermia and radiotherapy [185]. In contrast, another study suggested hyperthermia treatment 1–3 h after radiotherapy, and observed a high rate of complete response [186]. Cervical cancer patients treated with hyperthermia 1–4 h after radiotherapy also showed an improved complete response rate and three-year overall survival [187]. Conversely, some studies reported the effects of hyperthermia which was not concurrently combined with radiotherapy. Hyperthermia treatment twice per week and radiotherapy treatment five times per week increased complete response, but not the overall survival [188]. Another study that used hyperthermia as an irregular, non-scheduled additional treatment showed no beneficial effect on the suppression of locally advanced non-small-cell lung cancer [189]. One study emphasized that the delivery strategy of hyperthermia upon radiotherapy determined the therapeutic efficacy enhancement, which was supported by another study performed in various superficial cancer patients [181]. Research showed that the radiosensitization effect of hyperthermia was maximized when the therapies were concurrently administered. Although clinical research supported better outcomes with therapies combined with hyperthermia, some studies suggested the need to discover the underlying molecular mechanisms for better validation of hyperthermic effects [190]. Therefore, unveiling the biological factors involved in hyperthermia-induced radiosensitization could lead the way to a better understanding of the therapy.

The potential of hyperthermia immunogenicity was also validated via combining with other anti-tumor therapies. In gastrointestinal, and head and neck cancer patients, hyperthermia induced chemosensitivity to certain drugs, including cisplatin and cetuximab, with promising outcomes [186,190]. Triple combination therapy with hyperthermia, radiotherapy, and chemotherapy was performed in a previous study that suggested better recurrence-free and overall survival rates [191]. The hyperthermia-induced chemosensitization effect was also shown in previous studies. This combination therapy could be performed through regional hyperthermia or heated anti-tumor drug infusion. This combination therapy significantly improved overall survival and progression-free survival in some types of cancer [192,193]. A study supported the combination of hyperthermia and chemotherapy induced immunogenicity through providing clinical results [194]. Furthermore, the immunogenicity of hyperthermia treatment improved immunotherapeutic efficacy in some clinical research. Head and neck cancer, and breast cancer patients treated with hyperthermia and immunotherapy showed significantly improved survival outcomes [195,196]. Although only a small amount of clinical research into the combination of hyperthermia and immunotherapy has been performed, this combination therapy is believed to be promising. Taken together, the immunogenic effects of hyperthermia treatment could also sensitize the tumor cells against other anti-tumor therapies. However, more investigation into combination therapy is required to achieve therapeutic utilization.

## 5. Conclusions

Enhancing therapeutic efficacy has been widely studied for many anti-tumor therapies. In this review, we focused on hyperthermia and its immunogenic effects to sensitize tumor cells against radiotherapy. Studies showed that hyperthermia-induced radiosensitization could occur through enhancing immunogenicity against tumor cells, and that sensitization was clinically significant. As hyperthermia-induced molecular events remain partially unknown, and combination therapy cannot completely eliminate the tumor, further studies are needed to optimize the strategies of combination therapies. Furthermore, we discussed the clinical significance of hyperthermia in combination with chemotherapy and immunotherapy, which suggests the promising potential of optimized combination therapy as an anti-tumor strategy.

To develop hyperthermia treatment as an optimal combination therapy with radiotherapy, we suggest devising optimized hyperthermia treatment protocols considering the immunogenic effects of hyperthermia needed. Although clinical studies showed the schedule of combination therapy dominantly determined the therapeutic outcomes, research into the best schedule design is scarce. Combining other anti-tumor therapies with hyperthermia and radiotherapy is also expected to improve clinical outcomes. Moreover, basic research to understand the elusive molecular events upon hyperthermia can help investigations.

Although the potential of immunogenic treatment in therapeutic sensitization has been suggested, the difficulty in the delivery of immunogenic stimulators, including chemical compounds and immune checkpoint blockades, and low efficacy, slowed clinical success. We suggested hyperthermia treatment as an alternative immunogenic treatment with better delivery and spatial selectivity. Furthermore, hyperthermia treatment elevated heat shock response proteins, which exerted immunogenic effects and marked thermally stimulated sites. Therefore, hyperthermia treatment can compensate for the weak parts of other therapies, and further advances in hyperthermia technology can be used for technical improvement of therapeutic delivery systems. Taken together, we anticipate that further basic, clinical, and technological research into combining hyperthermia and radiotherapy will show a new path to complete tumor control.

## Figures and Tables

**Table 1:** Signaling pathway and proteins induced by combination of hyperthermia radiotherapy.

Signaling Pathway	Protein	Reference
DNA repair	DNA-PK	[135,139,147]
	γH2AX	[139,144]
	MRE11	[140,141,142,143]
	Rad50	[143]
	NBS1	[143]
	Rad51	[145]
	BRCA2	[146]
	Ku70	[147]
Cell cycle/Apoptosis	p53	[149,157]
	Cyclin-B1	[151]
	HMGB1	[152,156]
	Bax/Bcl-2	[157,158,159]
	NF-κB	[160]
	Akt	[161]
Heat shock response	Hsp90	[155]
	Hsp70	[156,167]
	Hsp72	[164,168]

## Abbreviations

Angpt	angiopoietin;
ATM	ataxia telangiectasia mutated
BRCA	breast cancer gene
CTLA-4	cytotoxic T-lymphocyte-associated protein 4
CXCL	chemokine (C-C motif) ligand
DC	dendritic cell
DNA-PK	DNA-dependent protein kinase
FDA	Food and Drug Administration
HMGB1	high motility group box protein B1
HSF	heat shock transcription factor
Hsp	heat shock protein
ICAM	intracellular adhesion molecule
IFNγ	interferon gamma
iNOS	inducible nitric oxide synthase
NF-κB	nuclear factor kappa beta
NK	natural killer
NKG2D	natural killer lectin-like receptor gene 2D
NO	nitric oxide
PD-1	programmed death 1
PD-L1	programmed death-ligand 1
TLR	toll-like receptor
TNF-α	tumor necrosis factor α
VCAM	vascular cell adhesion molecule
VEGF	vascular endothelial growth factor
